# Optimal Ways of Safflower Oil Production with Improvement of Press Equipment

**DOI:** 10.3390/foods13121909

**Published:** 2024-06-17

**Authors:** Mukhtarbek Kakimov, Maigul Mursalykova, Bożena Gajdzik, Radosław Wolniak, Gulnara Kokayeva, Michał Bembenek

**Affiliations:** 1 Department of Technology of Food and Processing Industries, S. Seifullin Kazakh Agro Technical University, Astana 010011, Kazakhstan; muhtarbek@mail.ru; 2 Department of Industrial Informatics, Silesian University of Technology, 44-100 Gliwice, Poland; 3 Faculty of Organization and Management, Silesian University of Technology, 44-100 Gliwice, Poland; rwolniak@polsl.pl; 4 Department of Technological Machinery and Equipment, S. Seifullin Kazakh Agro Technical Research University, Astana 010011, Kazakhstan; g.kokaeva@kazatu.edu.kz; 5 Faculty of Mechanical Engineering and Robotics, AGH University of Krakow, A. Mickiewicza 30, 30-059 Krakow, Poland; bembenek@agh.edu.pl

**Keywords:** safflower oil, food production, screw press, pressing process, pressure-regulating mechanism, product quality, comprehensive evaluation

## Abstract

This study aims to improve press equipment for safflower oil production by using a mechanism that optimizes pressure distribution within screw turns. A detailed analysis of the main components of the produced safflower oil was performed, encompassing both quantitative and qualitative assessments. Through the exploration of dependencies governing the safflower oil pressing process on the screw press, the optimal parameters were determined. As a result of the research, the optimal diaphragm gap between the gape cylinder and the pressing screw was determined, with the optimal oil yield percentage achieved at ω = 6.2 rad/s and δ = 5 mm. The study also compared the performance of the existing Dream Modern ODM-01 screw press and its upgraded version by analyzing the extracted oil. The results reveal changes in the quantitative and qualitative composition of the main oil components following the operation of the existing and the modernized screw presses. For instance, the amount of unsaturated fatty acids, such as oleic acid (7.7 ± 0.566%), linoleic acid (85.3 ± 1.185%), and linolenic acid (1.2 ± 0.223%), increased. There was an increase in the presence of inorganic substances in safflower oil: iron (0.023 ± 0.031 mg/kg), phosphorus (0.086 ± 0.059 mg/kg), silicium (0.136 ± 0.075 mg/kg), and others. The findings of this study hold significant commercial value and offer promising prospects for global market implementation.

## 1. Introduction

Safflower (*Carthamus tinctorius*) is a plant whose beneficial properties have been known to man for thousands of years. Safflower seed is valued as a source of high-quality edible oil with two main unsaturated fatty acids: oleic and linoleic, which make up about 90% of its total fatty acid content [1,2]. This crop is grown in arid regions where sunflowersand other similar crops do not take root [3]. Safflower oil is a unique product of plant origin, whose chemical composition allows it to be used for medical and cosmetic purposes, as well as in food production [4]. Considering its biological values and rich composition of vitamins and phospholipids, safflower oil production is currently a high-priority task [5].

The technology of vegetable oil production involves various processes applied to the raw oilseed material. Mechanical processes play a significant role in this technology [6]. Such processes as cleaning the seeds of impurities, breaking and separating the fruit and seed coats from the embryo and endosperm (kernel), grinding the kernel, and intermediate products of its processing, are mainly mechanical, preparing the material for intensive physical and chemical transformations [7].

In this context, our research team, having conducted comprehensive studies to improve safflower oil production technology, published the results of technical and technological solutions in scientific journals, approved by patents of the Republic of Kazakhstan for a utility model (№ 7977) and an invention (№ 36262) [8,9].

Significant contributions to the development of safflower oil production have been made by such groups of scientists asKhalid et al., Gibbins et al., Matthaus et al., Patrascoiuet al., and Al Surmi et al., and most scientific works focus on the nutritional value of safflower cultivation and preparation of safflower grain for processing [10,11,12,13,14]. There is less research on the technical and technological peculiarities of safflower processing than on other oilseeds. Almost all studies underline the future value of safflower crops and describe ways of preparing the crop for cultivation and processing. However, fewer works address the technical and technological specifics of safflower processing compared with other oilseed crops [15,16,17,18].

In vegetable oil production, pressing is one of the main methods used. Mechanical presses have been employed by various researchers to extract oil from different oilseed crops [19,20,21]. Many press designs are used in the industry for safflower oil extraction. For oil extraction, it is common to use screw or hydraulic presses in the case of oilseeds such as rapeseed [22], flax [23], sesame [24] sunflower [25,26,27], walnuts, pistachio nuts [28,29], and safflower seeds. Generally, screw presses offer some advantages over hydraulic ones, including higher oil yields and the capability for continuous or semi-continuous operation [30,31]. Additionally, oil extracted by a screw press is free of impurities [32].

Currently, there is no complete theory of screw press operation, and the design is mainly based on experimental studies and empirical data. This is because the properties of the pressed raw material change in the pressing channel, including density, size, particle size distribution, and oil content [33]. This is also due to the fact that the pressing pressure increases as the material moves towards the outlet. The scientific literature provides limited data on the performance of screw presses for safflower oil pressing and even fewer insights into the design of a working body with a diaphragm regulating mechanism for uniform pressure distribution during the pressing of vegetable oil in general. This often results in low oil yields and high power losses due to the inability to optimize pressure distribution effectively.

Some researchers [34,35,36] have presented results using a laboratory screw press with a regulating mechanism, typically used for juice production, which is rarely applied in vegetable oil extraction. Significant advancements in the pressing process have been made by scientists such as A. L. Kasenov et al., through extensive research over several decades [37,38,39].

In recent years, customer behavior has shifted significantly toward greater ecological awareness [40]. In light of all this, there is a need for a universal press capable of extracting oil from both low and high oil content crops for small-scale production. Our experimental equipment aims to accelerate the process of oil separation from safflower seeds without chemical methods, preserving the oil’s biological value, reducing costs associated with inter-operational transportation and labor, and increasing overall efficiency [41,42,43,44,45,46]. From this point of view, the present study aims to improve the screw press by developing a mechanism for uniform pressure distribution. This mechanism adjusts the pressure in the pressing chamber by narrowing the exit slot, thereby creating additional pressure.

## 2. Materials and Methods

### 2.1. Materials

#### Safflower Oil Extraction

**Figure 1:**
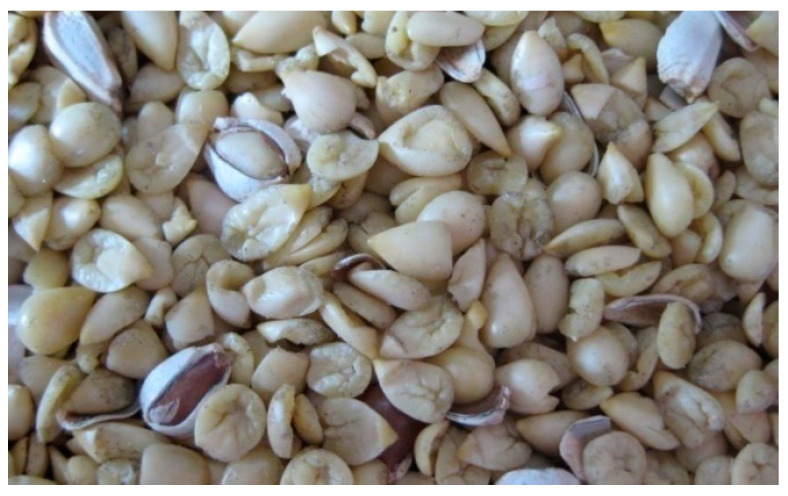
Cleaned safflower seeds.

The seeds of the safflower variety Akmai, obtained from samples in the World Safflower Collection at the Red Falls breeding station, were used for pressing [47]. This safflower variety (Figure 1) was grown in the Akmola region of Kazakhstan on experimental crops managed by the S. Seifullin Kazakh Agro Technical Research University, Astana, Kazakhstan. Akmai is the most popular safflower variety in Kazakhstan because of its high yield and resistance to diseases and climatic conditions. Consequently, it is widely grown in the Aktobe, Almaty, Zhambyl, Kyzylorda, South Kazakhstan, and Akmola regions. The fat content in fully dry seeds is 36–37%, while in kernels it is 55–55.7%, and the initial moisture is 5.8% [48].

**Figure 2:**
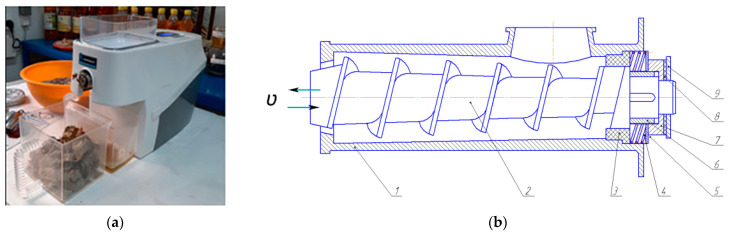
Experimental setup of the Dream Modern ODM-01 screw press (**а**) general view of the press equipment; (**b**) cross-sectional view of the press equipment: *υ*—progressive motion of the screw; 1—grain cylinder; 2—pressing screw; 3—sliding tip; 4—pressure regulator spring; 5—washer; 6—threaded nut; 7—lock nut; 8—rubber sealing ring; 9—control nut.

During the research on the safflower oil production process, the design of the working body of the Dream Modern ODM-01 screw press for processing this oilseed crop was modernized, as shown in Figure 2. The novelty of this technical solution, incorporated into the Dream Modern ODM-01 screw press (manufactured by RawMid, St. Petersburg, Russian Federation), is confirmed by patent № 7977 for a utility model granted by the Republic of Kazakhstan.

As shown in Figure 2, the experimental press consists of several components: a cone-shaped pressing screw, a zeer cylinder, a pressure control mechanism in the pressing chamber, a feed hopper, an oil-collecting tank, an electric motor, and a belt transmission. The press auger and zeer cylinder can be easily installed and removed, which makes it possible to keep the equipment sanitary and clean.

To control and monitor the power supply, a device is mounted on the surface of the rack for the monitoring of extraction process time, screw speed, temperature, and motor power. The primary innovation in the Dream Modern ODM-01 screw press for safflower oil production is the improved pressure control mechanism.

**Figure 3:**
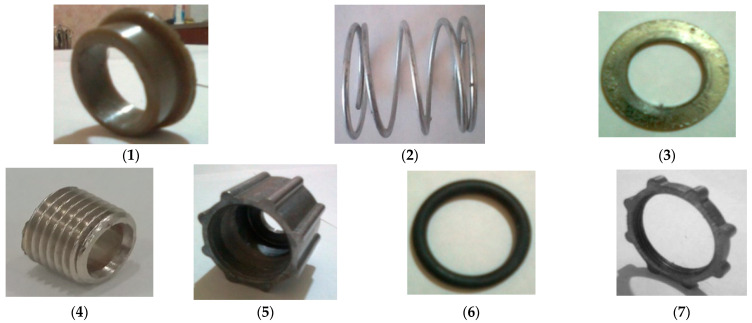
Pressure-regulating mechanism: (**1**)—sliding tip; (**2**)—pressure regulator spring; (**3**)—washer; (**4**)—screw nut; (**5**)—lock nut; (**6**)—rubber sealing ring; (**7**)—check nut.

In the Dream Modern ODM-01 screw press, the inner diameter of the press screw and the zeer cylinder is conical, with an inclination of 12°. This design feature allows the clearance between the pressure screw and the toothed cylinder to be adjusted by moving the screw forwards and backward. The pressure adjustment mechanism consists of a spring, a sleeve, an adjustment sleeve, two sliding tabs, a washer, and a rubber O-ring (Figure 3).

**Figure 4:**
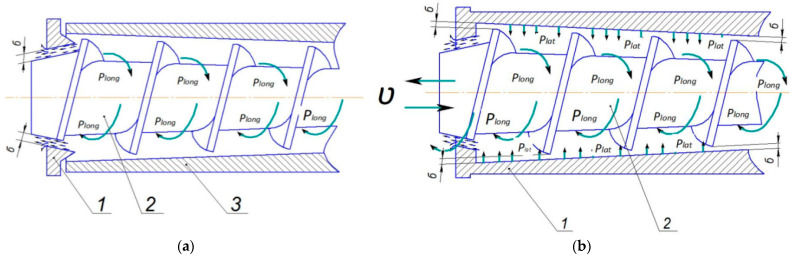
Diagram of pressure direction in the screw press using the standard approach (**a**) (*δ*—outlet opening narrowing due to pressure regulating mechanism; *P_long_*—longitudinal pressure created by the outlet opening; 1—pressure regulating cone; 2—pressure screw; 3—gape cylinder) and the proposed solution (**b**) (*P_lat_*—lateral pressure caused by narrowing of the gap between the screw and the gape cylinder; *υ*—direction of the pressing screw forward and backward movement; *δ*—adjustable gaps between the screw and the gape cylinder using the pressure adjustment mechanism; 1—gape cylinder; 2—pressing screw) pressure control mechanisms.

Figure 4a illustrates the pressure control mechanism of a conventional screw press. This mechanism creates additional pressure in the pressing chamber by narrowing the product outlet. By narrowing the outlet, the pressure is transferred to the product in the opposite direction through the winding channel, but this method cannot ensure uniform pressure distribution within the screw coils.

Since the product undergoes additional mass transfer during pressing, its structural and mechanical properties change in the cross-section with each turn of the screw. Initially, the bulk raw material is converted into a viscoplastic product, and by the end of the pressing, it becomes a semi-dry product. Such a complex structural and mechanical transformation of the raw material results in an unstable distribution of pressures in each turn of the screw, which prevents uniform pressure distribution. An unstable pressure surge can result in either insufficient or excessive pressure, failing to provide the optimal conditions needed for efficient oil extraction.

Therefore, this study is designed to implement a proposed pressure control mechanism where the pressing screw moves forwards and backward, as illustrated in Figure 4b. By moving the auger back and forth, the gap areas between the cylinder and the auger are narrowed based on the tapered outer and inner diameters of the auger coil. This adjustment creates the conditions for simultaneous contraction of each roll section and ensures uniform distribution of bilateral lateral and longitudinal pressure on the raw material.

With this proposed pressure control mechanism, pressure is applied simultaneously and in parallel along the longitudinal axis of the screw coils, rather than sequentially. Thus, the unilaterally transmitted pressure along the longitudinal axis reduces the load severalfold and limits the overpressure on the raw material being pressed. As a result, the oil yield is increased, the quality of the extracted oil is improved, the rapid wear of the working components is prevented, and overall efficiency increases.

The screw press operates as follows: cleaned safflower seeds are loaded into the feed hopper and enter the pressing chamber. As a result of the gradual narrowing of the press screw channel in the direction of product movement under the action of longitudinal pressure, oil flows out through the openings of the serrated cylinder into a special oil collection tank. The pressure in the pressing chamber is controlled by our proposed pressure control mechanism.

To understand the pressing process for safflower oil using a screw press, we conducted experimental studies based on the method described in the PhD thesis of Mursalykova M.T. (Republic of Kazakhstan, Semey, 2023) [49]. Our study involved:

- Investigating the influence of varying pressing speeds (ω = 5.2 rad/s; ω = 6.2 rad/s; ω = 7.2 rad/s; ω = 8.2 rad/s);
- Examining the impact of adjusting the initial and final diaphragm clearance (δ = 3 mm; δ = 5 mm; δ = 7 mm; δ = 9 mm) between the screw and the serrated cylinder on the pressing pressure.

The main objective of this research is to determine the factors that intensify the pressing process. By optimizing the speed and ensuring the appropriate pressure, we aim to achieve efficient oil separation. It is crucial to establish a balanced relationship between pressure and velocity and to understand the interdependencies of the diaphragm’s orifices. Therefore, we will analyze the parameters shown in the graphical data from our scientific-experimental work, providing an evaluation based on the relationships between velocities and diaphragm aperture sizes.

### 2.2. Methods

#### 2.2.1. Determination of Mass Fraction of Fat, Moisture, Protein, Ash, and Carbohydrates

The mass fraction of fat was determined using the Soxhlet method. This method involves multiple extractions of fat from a dried product sample with a solvent, followed by the removal of the solvent and drying of the fat to a constant weight. The extraction was performed using a Soxhlet apparatus, with petroleum ether used as the solvent (GOST 23042-86) [50].

The mass fraction of moisture was determined by drying the sample to a constant weight at a temperature of 103–105 °C (GOST 11812-2022) [51].

The mass fraction of protein was determined using the Kjeldahl method, which involves mineralizing the sample, distilling the ammonia into a sulfuric acid solution, and then titrating the sample (GOST 25011-2017) [52].

The mass fraction of ash was determined by ashing, a process that estimates the total mineral composition of the raw materials and finished product by burning the sample to a constant weight [53].

The mass fraction of the main carbohydrate was determined using the anthrone method, which involves heating monosaccharides with inorganic acids to convert them into furfural (oxymethylfurfural), which forms colored compounds with anthrone. The color intensity, measured colorimetrically, indicates the amount of carbohydrates present [54].

#### 2.2.2. Determination of Husk Content in Cleaned Kernels of Safflower Seeds

The preliminary preparation of safflower seeds is crucial for increasing the yield of safflower oil during the pressing process. One key factor to consider is the influence of the amount of husk in cleaned safflower seeds on the pressing process.

Preliminary separation of shells from the kernel enhances the oil content of the processed oilseed raw materials. When the low-oil components are removed, the relative oil content increases. This also improves the productivity of the technological equipment, as the working volume of the machines and apparatuses is not ballasted with low-oilseed material (the shells). Additionally, the quality of the oil improves because separating the shells prevents husk or husk lipids, which are rich in waxes and wax-like substances, from contaminating the oil. Their presence in the oil degrades its marketability by forming a fine suspension or “net” of small wax crystals, which can be removed only by prolonged oil treatment owing to their chemical inertness.

Another important aspect is that flaking the grain during the pressing process reduces the mechanical strength of its composition and prevents rapid wear of the working parts of the equipment, increasing its efficiency.

The optimum size and husk content in cleaned kernels of safflower seeds were determined according to GOST 12096-76 [55].

#### 2.2.3. Determination of Pressure Changes in the Pressing Process

The study of pressure changes was conducted following the methodology described in the thesis by Abdilova G.B for the degree of Candidate of Technical Sciences (Republic of Kazakhstan, Semey, 2010) [56]. MathCad 2000 (9.0) software was used for plotting the graphs, as well as JS Charts version 4.0.0—a graph and chart generator library thatallows for the creation of various types of charts, such as column charts, pie charts, and simple lines.

#### 2.2.4. Determination of the Energy Characteristics of the Plant

To determine the energy characteristics of plants, a measuring stand was constructed in the laboratory of the Department of Technological Equipment and Mechanical Engineering at the non-commercial joint stock company “Shakarim University of Semey City”. The measuring stand consists of a voltmeter, an ammeter, and a phasometer (a device for measuring “cos*φ*”). All the devices are integrated into the electric circuit that controls the electric motors of the press drive.

A method was developed to determine the energy characteristics of the experimental unit. It essentially involves calculating power based on the values of current, voltage, and cos*φ* measured directly by these devices. Safflower seeds were loaded into the hopper of the press so that the current, voltage, and cos*φ* could be measured. The electric motor was switched on, and the corresponding electrical values displayed by the instruments were recorded using a web camera connected to a computer. Subsequently, the measurement results were processed on the computer.

The same software and library as before were used for plotting and creating graphs—MathCad 2000 (9.0) and JS Charts.

#### 2.2.5. Determination of Physicochemical Parameters of Safflower Oil

Density and specific gravity were determined according to GOST 18848-2019 [57] on an AON-1 areometer from JSC “Steklopribor”. The refractive index of safflower oil was measured using a PAL-RI refractometer (Atago, Tokyo, Japan), following the method specified in GOST 3900-85 [58]. The density determination method consists ofimmersing the areometer in the test product, taking readings on the areometer scale at the determination temperature, and recalculating the results to the density at a temperature of 20 °C.

To measure the viscosity of safflower oil, a DV-E viscometer (Milwaukee, WI, USA) was used. Analyses were performed according to GOST 33768-2015 [59], which describes a method for determining the kinematic viscosity and calculating the dynamic viscosity of transparent and opaque liquids. The procedure essentially involves measuring the time of flow of a certain volume of the tested petroleum product under the influence of gravity using a glass capillary viscometer.

The iodine number, acid number, peroxide number, and saponification number of safflower oil were determined in accordance with AOAC [60].

#### 2.2.6. The Fatty Acid Composition of Safflower Oil

The fatty acid composition of safflower oil was determined according to GOST 30623-2018 [61] on an Agilent 7890A chromatograph (Agilent Technologies, Penang, Malaysia). High-performance liquid chromatography was used to measure the amount of α-, β-, γ-, and δ-tocopherols. The inorganic composition of pressed safflower oil was determined according to the relevant state standards of the Republic of Kazakhstan.

## 3. Results and Discussion

**Figure 5:**
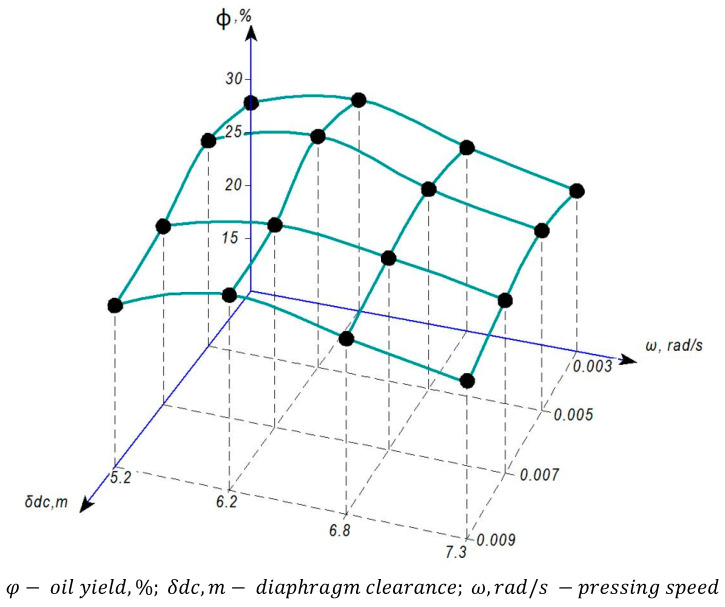
Dependence of safflower oil yield on speeds and apertures of the diaphragm in the pressing process.

**Figure 6:**
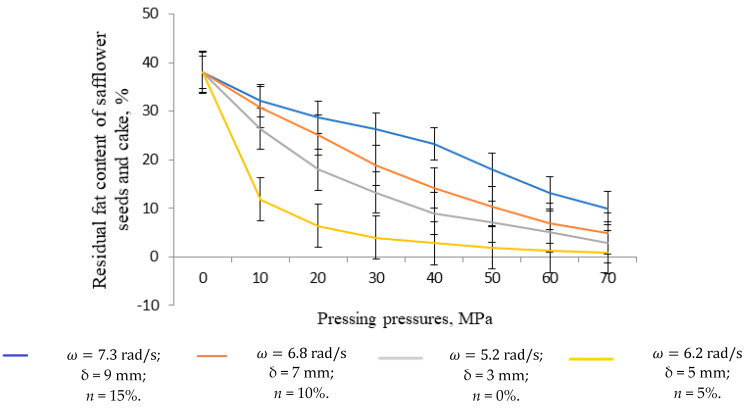
Dependence of the amount of residual fat in safflower cake during pressing on the speed and aperture of the diaphragm, as well as the percentage of husk content.

**Figure 7:**
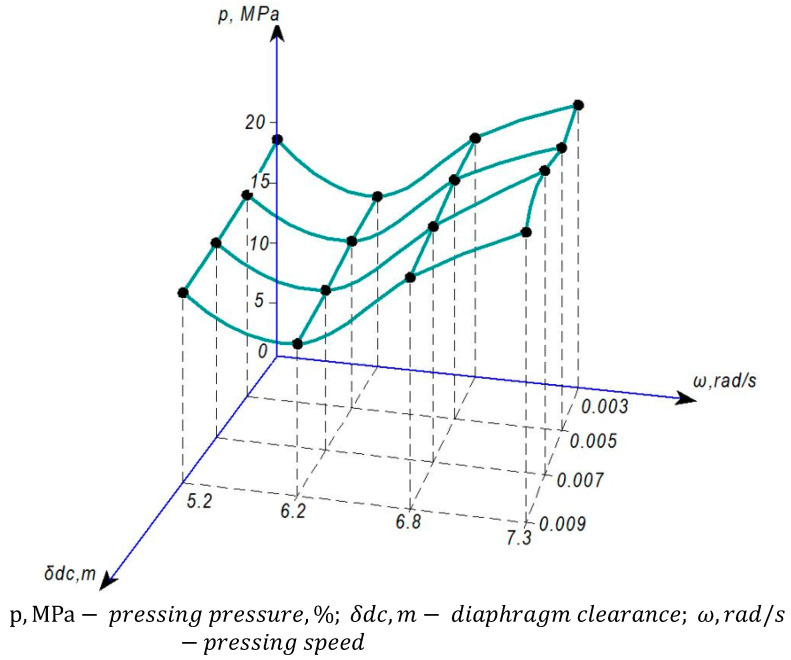
Variation of pressure, speed, and diaphragm clearance during the pressing of safflower seeds.

**Figure 8:**
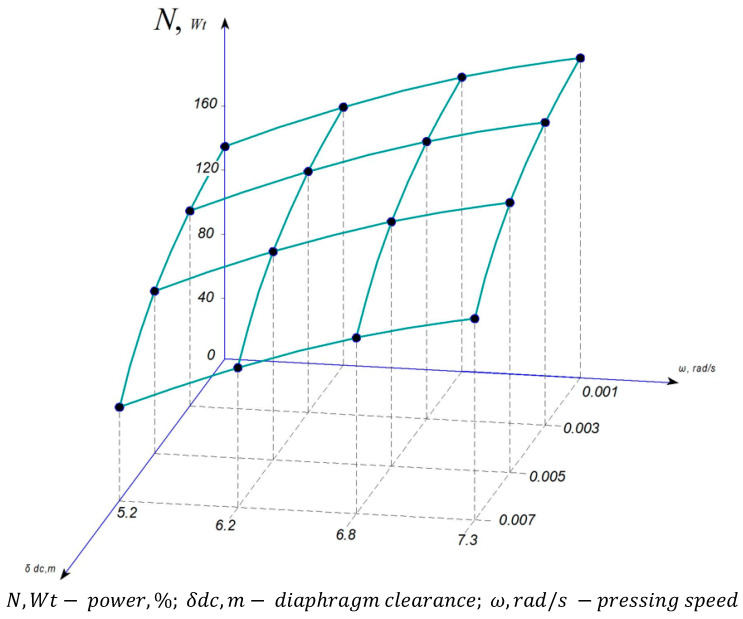
Dependence of pressing power on safflower oil yield and aperture of the diaphragm.

Experimental studies were conducted at the S. Seifullin Kazakh Agro Technical Research University and at the Shakarim University of Semey. As part of the improvement of the Dream Modern ODM-01 screw press equipment, an automatic pressure control mechanism was installed, marking the first implementation of such a mechanism in the working chamber of a screw press. The graphs shown in Figure 5, Figure 6, Figure 7 and Figure 8 are based on phenomena characterizing the efficiency of this automatic self-regulating mechanism. Consequently, we analyze changes in oil yield, pressing pressure, and equipment power as functions of screw speed and diaphragm clearances (the space between the screw and the zeer cylinder).

Table 1, Table 2 and Table 3 showcase the toxic elements and physico-chemical composition of safflower oil obtained, benefiting from the absence of overpressure losses during the pressing of safflower raw material.

The results hold significant commercial value and offer promising prospects for global markets. The practical application of these research findings will facilitate the mass processing and consumption of food products in Kazakhstan, as well as their export to other countries.

### 3.1. Investigation of Safflower Oil Yield in the Pressing Process

#### 3.1.1. Dependence of Safflower Oil Yield during the Pressing Process on Speeds and Diaphragm Clearances

The graph in *φ*−oil yield, %; δdc−diaphragm clearance, m; ω−pressing speed, rad/s.

Figure 5 shows that the maximum oil yield occurs when the diaphragm gap is set to δ = 5 mm. Beyond this point, narrowing the gap further leads to the mixing of other parts of safflower seeds with the released oil in the zeer cylinder, resulting in a change in the color of the oil to brown. At the same time, as the velocity increases, it can be observed that the differences in the variation of the aperture oil yield approach a minimum. This phenomenon can be attributed to the degradation of the pressing process due to the inability to separate oil from safflower seeds completely in a time-dependent manner at high speeds (ω = 7.2 rad/s, ω = 8.2 rad/s). Among the different velocities tested, the yield of safflower oil was found to be the lowest at a velocity of ω = 5.2 rad/s. Consequently, this velocity value is not considered a suitable parameter owing to the corresponding low oil yield and failure to comply with the performance indicator of the safflower oil technology standard. Therefore, we identify the velocity ω = 6.2 rad/s as the optimal structural parameter.

#### 3.1.2. Dependence of the Amount of Residual Fat Content in Safflower Cake during the Pressing Process on Speeds and Diaphragm Clearances and the Percentage of Husk Content

Pre-shearing of safflower plays a crucial role in facilitating an effective pressing process and ensuring high oil quality. Therefore, our research endeavors to prioritize addressing this critical aspect. The pressure applied by the screw presses is mechanically sustained, adequately separating fat accumulated in macrocapillaries but often leaving fat trapped under adsorptive conditions in microcapillaries. Under the action of adsorption and capillary forces within microcapillaries, the pressing pressure increases several times during pressing. As a consequence, complete oil extraction from safflower through pressing alone remains unattainable, since a portion of fat is left in the pressed oil cake.

Half of the pressing pressure is directed toward oil extraction, while the other half is directed toward processing lean (safflower husk) dry parts. The efficacy of the pressing process hinges on the movement of oil and fat within macrocapillaries under applied pressure. Whether more oil is released depends on its viscosity and the state of the raw material during plastic deformation, relative to its modulus of elasticity.

When safflower is heated, the sticky substances accumulated as a result of the action of hydrolyzed collagen adhere to the fine fat particles accumulated within the microcapillary, reducing its porosity at high temperatures. Adding a small amount of husk to soft grains suitable for pressing reduces the plasticity of the pressed safflower, enhancing its structural integrity and facilitating better oil distribution. Furthermore, safflower husk exhibits high strength capacity and acts as a drainage facilitator in fat production. Thus, supplementing safflower with husk can increase pressed oil yields by 1–2%.

Given these complexities, we analyze all multifactorial experimental studies regarding speed and aperture dependencies on husk percentage and present only the optimal results on the corresponding graph in Figure 6.

The graph in Figure 6 illustrates the minimum residual fat content in safflower cake achieved at a speed of ω = 6.2 rad/s and an aperture gap of δ = 5 mm, along with a husk content percentage of N = 5%. As the percentage of husk content increased beyond N = 10%, several adverse effects were observed, including a decrease in oil yield, operation of the equipment under high voltage, a sharp increase in required power, and a change in the color of the released oil to dark brown. Therefore, we concluded that the effective moisture in safflower cake should ideally fall within the range of 3–5%.

Furthermore, at a pressure of P= 30 MPa with the pressure control mechanism engaged, the residual fat content in safflower cake reached its lowest level. This indicates that further increases in pressing pressure produce no significant benefits.

In summary, we have found that the pressing pressure necessary for optimal oil extraction can be achieved by adjusting the relationships between aperture orifices and screw speeds. However, we have also noticed that maximizing the speed and narrowing the diaphragm clearances did not provide the optimum pressure for extracting the oil. Although high pressure was supplied, it is still important to incorporate an automatic pressure control mechanism during the pressing process.

Theoptimal values leading to parametric dependencies were determined at a velocity ω = 6.2 rad/s, an aperture gap of δ = 5 mm, and a husk percentage of N = 5%. These indicators were not randomly chosen and can be explained by the following patterns:

1. At screw speeds of ω = 7.2 rad/s and ω = 8.2 rad/s, the residual fat content in safflower cake is higher compared with speeds of ω = 6.2 rad/s and ω = 5.2 rad/s. This is due to the incomplete separation of oil from safflower grain over time. Hence, the low screw rotation speeds of widely used screw press equipment (e.g., the ‘Atlas’ press, France: 1.5 rad/s, the B6-FOA press: 6.8 rad/s, the E8-FOB press: 0.2–2.09 rad/s, the ETR-20 press: 1.25 rad/s, the MP-68 press: 1.8 rad/s, 2.5 rad/s, 3.8 rad/s, etc.). We select the screw speed of ω = 6.2 rad/s as optimal because of the superior physical and chemical properties of the compressed oil compared with the speed of ω = 5.2 rad/s.
2. The diaphragm clearance (adjustable clearance between the auger and gape cylinder) of δ = 5 mm was chosen as the optimal index. During experimentation, we attempted to narrow the aperture opening as much as possible, but narrowing it further to δ = 3 mm did not yield favorable results, instead leading to the deterioration of the quality of the released oil, a change in the color, and the equipment operating at higher voltages.
3. A husk percentage of N = 5% was determined to be optimal. Adding husks to the pressed raw material in small quantities during the pressing process reduces its plasticity, improves its structure, and aids in fat release. Using this value resulted in a 1–2% increase in pressed oil content.

### 3.2. Investigation of Pressure Changes during the Pressing Process

One of the most important parameters in studying the pressing process is the pressing pressure. Depending on the complexity of the installation for each number of rolls, a pressure-sensing device is installed at the product outlet, considering the structural condition of the press equipment. Additionally, based on theoretical equations, the pressure difference between the press screw rolls was determined, taking into account the structural and mechanical properties of the samples taken from each roll, as illustrated in Figure 7.

As mentioned earlier, in a conventional screw press, the pressure in the pressing chamber is controlled by a conical orifice at the product outlet. At that time, the product is stuck in the outlet opening, leading to pressure distribution in the press channel in reverse flow, i.e., by the longitudinal pressure. This instability in pressure values occurs because of changes not only in the volume of the product but also in its mass and phase composition, resulting in fluctuating pressure levels that are suboptimal for the pressing process.

Upon pressure determination, as depicted in Figure 7, two general phenomena can be observed: an increase in pressure due to a decrease in fat content in safflower cake, and an increase in velocity. These phenomena can be attributed to two distinct reasons:

1. Structural changes in processed raw materials occur through oil separation.
2. The flow of processed raw materials increases under the influence of velocities.

The impact of speed on pressing pressure outweighs that of the pressing process itself. Figure 8 shows that despite the deterioration of the pressing process with increasing speed, i.e., increasing the percentage of fat content in safflower cake, the pressure also increases.

At higher velocities, the impact of pressures intensifies, causing the orifice differentials of the diaphragms to diverge. This phenomenon arises from the heightened percentage of oil in the aperture openings due to increased pressing speed, reduced viscosity, and consequent augmented flows.

Therefore, based on the experimental findings, the following conclusion can be drawn: merely increasing pressing pressure does not always ensure a decrease in oil content within the pressed product. Instead, determining the optimal pressure value involves experimental assessment, taking into account the technological intricacies and structural and mechanical properties of the product, as well as the quantity and quality of the resulting oil. Accordingly, the pressure in the press equipment requires continuous monitoring and adjustment.

### 3.3. Energy Characteristics of Experimental Press Equipment

During the experiment, the energy characteristics of the pressing process and the fluctuation in equipment power relative to pressing speed and diaphragm clearance are outlined, as presented in Figure 8.

Figure 8 suggests that there is a direct connection between increased oil yield and higher power consumption. This relationship can be attributed to the fact that the increase in external and internal friction leads to an increase in required power resulting from the structural and mechanical alterations induced by oil release.

The experimental results indicate that the power increases along with the velocity and narrowing of the aperture openings of the diaphragm. It is known that this phenomenon is characteristic of all processes. However, at each pressing speed, the power value varies depending on the influencing factors. It can be observed that the differences in power indices at the aperture openings become tighter owing to the increase in velocities. This phenomenon can be explained by the increase in pressure due to the increase in velocity.

### 3.4. Physical and Chemical Parameters of Safflower Oil

We determined various physical parameters of safflower oil such as density, refractive index, moisture content, and volatile matter content. These results are summarized below in Table 2.

From the results of Table 2, it can be seen that the density of safflower oil after using the existing screw press was 0.924 g/cm^3^. After utilizing the modernized screw press, a slight decrease in density to 0.919 g/cm^3^ can be observed. The density of a substance is the ratio of its mass to its volume, while specific gravity is the ratio of the mass of a certain volume of a substance to the mass of the same volume of water at 20°C. Both density and specific gravity are temperature-dependent. The specific gravity of vegetable oils is also influenced by the quantitative content of polyunsaturated fatty acids. For example, the C_18_ ratio of polyunsaturated fatty acids in safflower oil can significantly affect its specific gravity. Thus, the specific gravity of safflower oil after using the existing screw press was 0.929, and with an increased proportion of polyunsaturated fatty acids, it rose to 0.962. While the determination of the specific gravity and refractive index of the oil does not provide sufficient information for quantitative identification purposes, these data are useful for detecting adulteration and contamination of the oil [62].

The more linoleic and linolenic acids, which belong to the group of polyunsaturated fatty acids, are present in the oil, the higher its refractive index. The refractive index also reflects the oil’s quality, purity, and degree of oxidation during storage. The refractive index of safflower oil with the existing screw press was 1.445, and with the modernized screw press, it also increased to 1.466. The viscosity of the safflower oil showed a slight change: with the existing screw press, it was 46.4 cP, and with the modernized screw press, it was 46.9 cP.

Additionally, the iodine number, acid number, saponification number, and peroxide number of safflower oil were determined, with the results displayed in Table 3. These measurements provide insights into the chemical composition and quality of the oil.

To analyze some of the parameters, we have used the ANOVA test. In the case of pressure characteristics, the ANOVA test yields values of H(3, *n* = 16) = 9.51, *p* = 0.023. The *p*-value of 0.023 is less than the significance level α=0.05, suggesting that there are statistically significant differences in pressure characteristics among the groups studied. This implies that the pressure exerted during the oil extraction process varies significantly depending on the group, which could be attributed to the different types of screw presses or the settings used during the extraction.

The test reveals that the pressure applied during the extraction process is not uniform across different groups. This could be due to variations in the design or operation of the screw presses used, or differences in the settings such as speed or diaphragm clearances.

The acid number represents the amount of potassium hydroxide (KOH) in milligrams required to neutralize free fatty acids in the oil. According to the data in Table 3, the acid number of safflower oil after using the existing screw press was 1.07 mg KOH/g, while after using the modernized screw press, the acid number slightly decreased to 1.065 mg KOH/g.

The iodine number indicatesthe degree of unsaturation of fatty acids in oils. The iodine number of safflower oil with the existing screw press was 144.19 g I_2_/100 g, whereas with the modernized screw press, it was 147.15 g I_2_/100 g, which confirms the increase in the amount of unsaturated fatty acids.

The peroxide number after using the existing screw press was 8.07 mol/kg, and after using the modernized screw press, it became 7.65 mol/kg, which is a good indicator of storability.

The saponification number represents the amount of KOH required to saponify the fat and indicates the ester bond content. The saponification number of safflower oil with the existing screw press is 160.4 mg KOH/g, and with the modernized screw press, it is 163.8 mg KOH/g.

### 3.5. Fatty Acid Compositionof Safflower Oil

The results of the studies on the fatty acid composition of oil produced using the existing and modernized screw presses are presented in Table 4.

In the case of safflower oil yield, the ANOVA test results are as follows: H(3, *n* = 16) = 10.01, *p* = 0.018. The ANOVA test is significant at the statistical significance level of α = 0.05. The results for the safflower oil yield differ among the various groups studied. The *p*-value of 0.018 is also below the significance threshold of α=0.05, indicating statistically significant differences in the oil yields between the different groups. This suggests that the yield of safflower oil varies significantly based on the type of screw press or the specific parameters used in the extraction process.

The test shows that the amount of oil extracted differs significantly among the groups. Factors contributing to this variation could include the efficiency of the screw press, the condition of the seeds, or specific operational parameters.

The findings are crucial as they suggest that optimizing the extraction process by selecting the appropriate screw press and adjusting its settings can significantly impact both the pressure characteristics and the yield of safflower oil. This could lead to improvements in the efficiency and cost-effectiveness of the oil extraction process.

Following the analysis of the fatty acid composition of safflower oil, several conclusions can be drawn. For saturated fatty acids, C_18:0_ stearic acid in safflower oil produced by the existing screw press was identified at 2.2%, whereas the modernized screw press produced safflower oil with the amount of stearic acid reduced to 2.0%.

From Table 4, it is evident that there is a quantitative advantage of unsaturated fatty acids over saturated fatty acids. Notably, after the use of the modernized screw press, the concentration of unsaturated fatty acids increased significantly, although there are exceptions where the content of individual fatty acids decreased.

**Figure 9:**
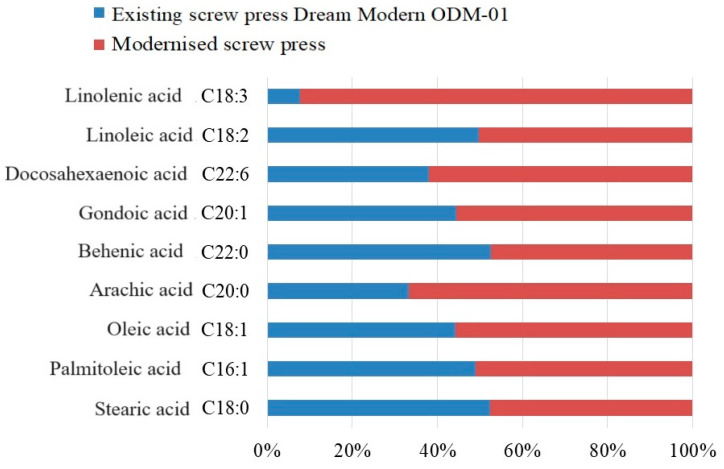
Fatty acid composition of pressed safflower oil.

Focusing on each group of unsaturated fatty acids, we consider monounsaturated and polyunsaturated fatty acids separately:

- Monounsaturated fatty acids: The content of C_16:1_ palmitoleic acid (5.1%), C_20:0_ arachic acid (0.8%), C_18:1_ oleic acid (7.7%), C_22:0_ behenic acid (0.27%), C_20:1_ gondoic acid (0.25%), and C_22:6_ docosahexaenoic acid (2.6%) increased (Figure 9).
- Polyunsaturated fatty acids: These predominate when using both the existing and upgraded screw presses. For example, the C_18:2_ linoleic acid content increased from 84.2% with the existing screw press to 85.3% with the upgraded screw press. The concentration of C_18:3_ linolenic acid increased slightly from 0.1% to 1.2%. These results align closely with those reported by other authors [63,64].

The inorganic composition of safflower oil also changed significantly after using the upgraded screw press, with the concentration of many elements greatly increasing. However, although the amount of sulfur was 0.072 mg/kg in the case of the standard screw press, after using the upgraded one, it was not detected in the safflower oil composition. As for the other inorganic substances, the content of iron increased from 0.018 to 0.023 mg/kg, the content of phosphorus—from 0.076 to 0.086 mg/kg, that of silicium—from 0.054 to 0.136 mg/kg, that of chlorine—from 0.087 to 0.325 mg/kg, and that of calcium—from 0.097 to 0.178 mg/kg, at least doubling in each case.

The obtained research results were considered in comparison with the results obtained using modern pressing equipment [65,66,67,68,69]. According to the results obtained, the graphs shown in Figure 5, Figure 6, Figure 7 and Figure 8 were plotted and the parameters characterizing the efficiency of the self-regulating mechanism were substantiated. The optimum safflower oil yield was determined depending on the pressing speed and the diaphragm gap (the gap between the screw and the serrated cylinder), as well as the density and structural–mechanical properties of the product, pressing pressure, and energy characteristics of the press were determined.

**Figure 10:**
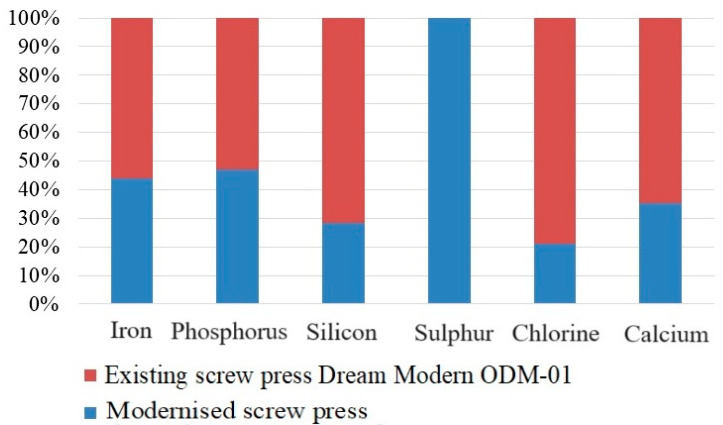
Inorganic composition of pressed safflower oil.

Table 2, Table 3 and Table 4 and Figure 9 and Figure 10 show the comparative quantitative and qualitative composition of safflower oil in comparison with the existing equipment.

As a result of the research, the following indicators were achieved in comparison with the indicators obtained by using modern equipment for pressing: an increase in oil yield of 0.3%, reduction inenergy consumption for pressing pressure by almost two times, reduction inmass fraction of non-fatty impurities of 0.02% and acid number of 1.060 mgКON/g, reduction inperoxide number to 7.01 mmol/kg 1/2O and the amount of toxic elements by two times, and an increase inantioxidant activity of 42.65%. As a result, the quality of safflower oil improved and its shelf life increased from 6 to 12 months.

At the introduction of the received results in technology of safflower oil production, the following consumer and economic effects can be expected:

- improved product quality—allowingfor more precise control of the pressing process, which affects the production of high-quality safflower oil;
- increased productivity—through the use of a self-regulating pressure mechanism;
- resource optimization—efficient use of equipment capacity and workforce;
- reduction inproduction time—due to intensification of the pressing process;
- improving product safety—reducing oil loss during pressing, improving oil quality, and ensuring food safety;
- cost savings—reduces costs for self-regulating mechanisms, maintenance, and quality control, i.e., optimizes processes and resources and reduces production costs.

Thus, the research was fully conducted, but more research is needed in the future to determine the rheological properties of the products, as the implementation of the findings into production will bring significant benefits to production companies and society as a whole.

The obtained results have a high commercial component and good prospects for realization on the world markets. The ecological effect is the development of knowledge-intensive technologies in safflower oil production, in particular in obtaining natural and environmentally friendly safflower oil. The social effects of the results obtained will contribute to the development of Kazakhstan in the vegetable oil industry, increase the efficiency of rational use and diversification of energy resources, and contribute to the further development of alternative methods. The practical application of the research results will create favorable conditions for mass production and consumption of food products in Kazakhstan, as well as their export to other countries. Therefore, the practical application of the results obtained with approbation in production is considered in the future. Also, the feasibility of the proposed self-regulating mechanism will be further explored for other presses used in food production.

## 4. Conclusions

Vegetable oils, including safflower oil, are currently gaining popularity as products used for both food and technical purposes. For the human body, the nutritional value of safflower oil is very high, especially for the prevention and treatment of various diseases.

One of the main processes used in the production of vegetable oil is the pressing process. It is one of the most important stages of production technology, as it affects the quality of the oil and its shelf life.

The study established the mechanism of self-regulating pressure in screw pressesand experimentally determined the optimal parameters of pressing pressure from the dependent factors. The composition of the main components of safflower oil was analyzed, the quantitative and qualitative compositions were determined, and a comprehensive assessment of the oil obtained by pressing in the existing and modernized equipment was given.

As a result of the research, the following indicators were achieved in comparison with the indicators obtained by using modern equipment for pressing: an increase in oil yield by 0.3%, reduction inenergy consumption for pressing pressure by almost two times, reduction in themass fraction of non-fatty impurities by 0.02% and acid number by 1.060 mgКON/g, reduction inperoxide number to 7.01 mmol/kg 1/2O and the amount of toxic elements by two times, and an increase in antioxidant activity of 42.65%. As a result, the quality of safflower oil improved and its shelf life increased from 6 to 12 months.

Thus, the obtained results provide consumer and economic effects when implementing modernization in safflower oil production technology, such as improving product quality, increasing productivity, optimizing resources, reducing production time, improving product safety, and reducing costs. In addition, the obtained research results could contribute to further commercialization and are promising for implementation in world markets. The use of modernized equipment in the production of safflower oil will create favorable conditions for mass production of this product in Kazakhstan, as well as its export to other countries.

## 5. Patents

Mursalykova Maygul Taurzhanovna, Kakimov Mukhtarbek Mukanovich, Kassenov Amirzhan Leonidovich, and Iskakov Bauyrzhan Myrzabekovich. Oil extraction screw press. For utility model patent No. 7977, filed 12 January 2023, and issued 21 April 2023.

Iskakov Bauyrzhan Myrzabekovich, Kakimov Mukhtarbek Mukanovich, Tokhtarov Zhaiyk Khamitovich, Mursalykova Maygul Taurzhanovna, and Sataeva Zhuldyz Isakovna. Centrifuge for deep purification of vegetable oils from mechanical impurities. For invention patent No. 36,262, filed 9 March 2022, and issued 15 September 2023.

## Figures and Tables

**Table 1:** Technical specification.

General Characteristics	Dream Modern ODM-01 Screw Press	Modernized Screw Press
Rated power	200 Watt	
Thermal capacity	120 Watt	
Rotational speed	50 rpm	50–80 rpm
Pressure adjustment mechanism	no	yes
Clearance between zeer cylinder and screw	0.003 m	0.003 m to 0.009 m
Type of control	electronic	
Temperature regime	30–180 °C in 10-degree increments	
Weight and dimensions:		
Dimensions of the assembled equipment	0.41 m × 0.28 m × 0.18 m	
Weight of equipment	5.5 kg	5.7 kg

**Table 2:** Physical parameters of safflower oil.

Physical Indicators			
№	Indicator	Dream Modern ODM-01 Screw Press	Modernized Screw Press
1	Density (g/cm^3^)	0.924 ± 0.192 *	0.919 ± 0.195 *
2	Specific gravity	0.929 ± 0.196 *	0.962 ± 0.2 *
3	Refractive index	1.445 ± 0.245 *	1.466 ± 0.247 *
4	Viscosity (cP)	46.4 ± 1.39 *	46.9 ± 1.397 *

* Each value is the average of three definitions ± standard deviation.

**Table 3:** Physical parameters of safflower oil.

Chemical Parameters			
№	Indicator	Dream Modern ODM-01 Screw Press	Modernized Screw Press
1	Acid number (mg KOH/g)	1.07 ± 0.211 *	1.065 ± 0.21 *
2	Iodine number (g in I_2_/100 g)	144.19 ± 2.45 *	147.15 ± 2.47 *
3	Peroxide number (mol/kg)	8.07 ± 0.57 *	7.65 ± 0.56 *
4	Saponification number (mg KOH/g)	160.4 ± 2.58 *	163.8 ± 2.61 *

* Each value is the average of three definitions ± standard deviation.

**Table 4:** Fatty acid composition of safflower oil.

Name of Indicator	Characteristics and Norms, %	Dream Modern ODM-01 Screw Press, %	Modernized Screw Press, %
Saturated fatty acids			
Stearic acid C_18:0_	1.0–10.0	2.2 ± 0.302 *	2.0 ± 0.288 *
Monounsaturated fatty acids			
Palmitoleic acid C_16:1_	up to 0.5	4.9 ± 0.451 *	5.1 ± 0.461 *
Oleic acid C_18:1_	7.0–12.2	6.1 ± 0.504 *	7.7 ± 0.566 *
Arachic acid C_20:0_	up to 2.5	0.4 ± 0.129 *	0.8 ± 0.182 *
Behenic acid C_22:0_	up to 0.5	0.3 ± 0.111 *	0.3 ± 0.106 *
Gondoic acid C_20:1_	unregulated	0.2 ± 0.091 *	0.3 ± 0.102 *
Docosahexaenoic acid C_22:6_	unregulated	1.6 ± 0.258 *	2.6 ± 0.329 *
Polyunsaturated fatty acids			
Linoleic acid C_18:2_	55.0–81.0	84.2 ± 1.873 *	85.3 ± 1.185 *
Linolenic acid C_18:3_	up to 1.0	0.1 ± 0.064 *	1.2 ± 0.223 *
Inorganic composition: (mg/kg, not more)			
Iron	5.0	0.018 ± 0.027 *	0.023 ± 0.031 *
Phosphorus	unregulated	0.076 ± 0.056 *	0.086 ± 0.059 *
Silicium	unregulated	0.054 ± 0.047 *	0.136 ± 0.075 *
Sulfur	unregulated	0.072 ± 0.054 *	0.000
Chlorine	unregulated	0.087 ± 0.060 *	0.325 ± 0.116 *
Calcium	unregulated	0.097 ± 0.063 *	0.178 ± 0.086 *

* Values are expressed as arithmetic mean ± standard deviation.

## Data Availability

Data is contained within the article.
